# Characterising the Behaviour of a Structured PDE Model of the Cell Cycle in Contrast to a Corresponding ODE System

**DOI:** 10.1007/s11538-025-01472-8

**Published:** 2025-06-08

**Authors:** Ruby E. Nixson, Helen M. Byrne, Joe M. Pitt-Francis, Philip K. Maini

**Affiliations:** 1 Wolfson Centre for Mathematical Biology, University of Oxford, Oxford, UK; 2 Ludwig Institute for Cancer Research, University of Oxford, Oxford, UK; 3 Department of Computer Science, University of Oxford, Oxford, UK

**Keywords:** Cell cycle, Mathematical modelling, Structured PDE, Age-structured models

## Abstract

Experimental results have shown that anti-cancer therapies, such as radiotherapy and chemotherapy, can modulate the cell cycle and generate cell cycle phase-dependent responses. As a result, obtaining a detailed understanding of the cell cycle is one possible path towards improving the efficacy of many of these therapies. Here, we consider a basic structured partial differential equation (PDE) model for cell progression through the cell cycle, and derive expressions for key quantities, such as the population growth rate and cell phase proportions. These quantities are shown to be periodic and, as such, we compare the PDE model to a corresponding ordinary differential equation (ODE) model in which the parameters are linked by ensuring that the long-term ODE behaviour agrees with the average PDE behaviour. By design, we find that the ODE model does an excellent job of representing the mean dynamics of the PDE model within just a few cell cycles. However, by probing the parameter space we find cases in which this mean behaviour is not a good measure of the PDE population growth. Our analytical comparison of two caricature models (one PDE and one ODE system) provides insight into cases in which the simple ODE model is an appropriate approximation to the PDE model.

## Introduction

**Fig. 1:**
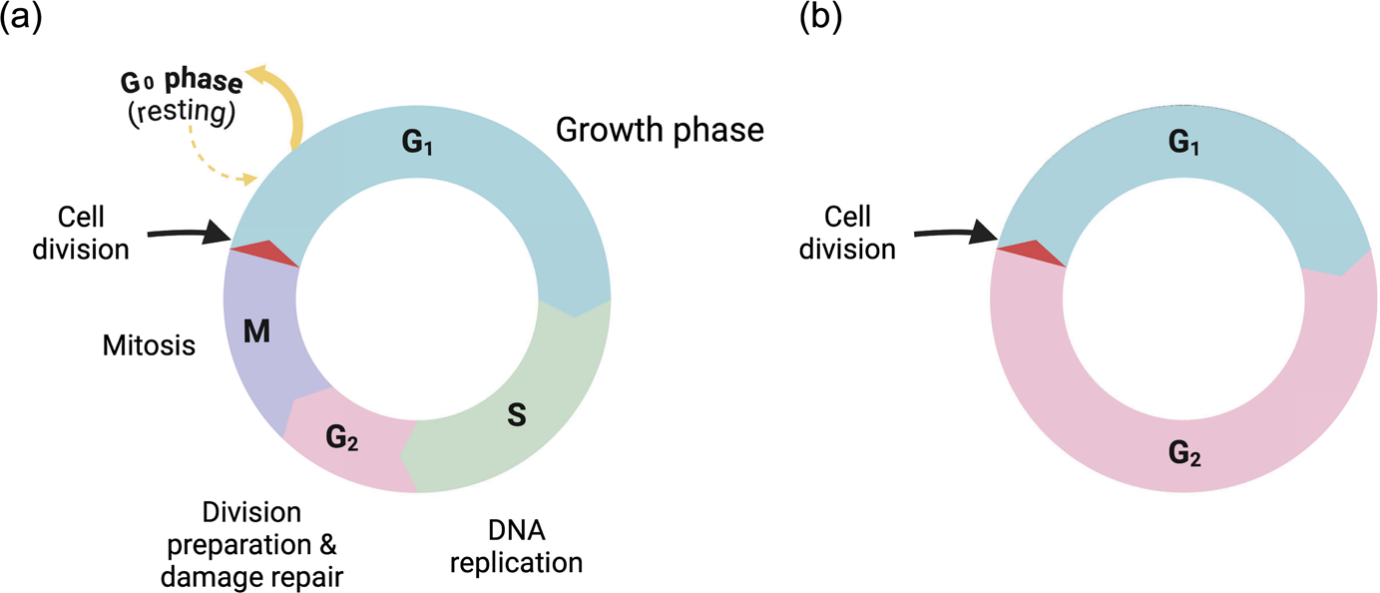
(a) A simple schematic illustrating the four phases of the cell cycle, along with the “quiescent" $\hbox {G}_0$ phase. (b) A simplified schematic of the cell cycle in which the S, $\hbox {G}_2$ and M phases are grouped together and labelled $\hbox {G}_2$. This figure was created with BioRender.com.

The cell cycle controls the duplication of cellular content and the eventual division of a cell into two daughter cells. A cell must pass through the four phases of the cell cycle before cell division takes place (see Figure 1). During the first phase, denoted by $\hbox {G}_1$, the cell faces a “choice" of either remaining in an actively cycling state or pausing cell cycle progression (Matthews et al 2022). Following this, the cell genome is duplicated in the S phase. This process is highly regulated to ensure that DNA duplication only occurs once per cycle (Matthews et al 2022). The cell then moves into the $\hbox {G}_2$ phase, followed by the M phase, where mitosis takes place. At the end of the M phase, cells divide to produce two identical daughter cells, and the cycle starts again. Throughout the cell cycle, complex regulatory pathways (Matthews et al 2022; Williams and Stoeber 2012; Liu et al 2022; Suski et al 2021) control the progression rate to the next phase.

Mathematical modelling of the cell cycle is an active field of research, spanning a variety of approaches, including agent-based models (ABMs), partial differential equations (PDEs) and ordinary differential equations (ODEs), with many of these models being applied to model cancerous cell populations. Global estimates indicate that 19.3 million new cancer cases were diagnosed and 10 million cancer deaths were reported in 2020 (Sung et al 2021). Mutations in cancerous cells alter the regulatory mechanisms found in the cell cycle of normal cells to benefit the survival and growth of tumours (Matthews et al 2022). Experimental results show that cellular responses to radiotherapy can be affected by cell cycle phase (Pawlik and Keyomarsi 2004; Lonati et al 2021; Muz et al 2015; Yashar 2018; Lonati et al 2021). Furthermore, chemotherapies for cancerous cells are also known to interact with the cell cycle, with some chemotherapeutic drugs modulating progress through the cell cycle, while others induce a cell cycle phase-dependent response (Schwartz and Shah 2005; Sun et al 2021; Otto and Sicinski 2017). A review of mathematical modelling of the cell cycle and its impact on anti-tumour treatment strategies is provided by Ma and Gurkan-Cavusoglu (2024).

The use of structured population models, in which the cell population is divided with respect to some important property, for example, time spent in a certain cell cycle phase (see Caswell et al 1997 for a general overview of structured population models), has been explored in multiple ways. In early work, Rubinow (1968) presents a structured PDE model of actively-cycling cells, where cell cycle progression is used to structure the population. The speed with which the progression variable changes is assumed to depend on the current cell cycle position, and an analytical solution for the density of cells at a given progression level and time is derived. This model forms the basis for the work in this paper.

Much of the recent work on cell cycle modelling focuses on stochasticity and computational techniques, such as numerical solutions to PDE systems or implementation of ABMs (see Kynaston et al 2022; Jin et al 2021; Celora et al 2024), while the analytical study of the ODE and PDE models is not so common. A comparison of three increasingly-complex ODE systems and a structured PDE model was recently conducted by Ubezio (2024). The PDE model has cell populations structured by the time which they have spent in their current cell cycle phase, where cells progress through the cell cycle phases at fixed time intervals. Furthermore, cell death and quiescence is considered. The complexity of the proposed PDE model means that analytical results are not considered; instead each of the four models are fitted to experimental data. The fit to experimental data is found to be much improved when the models are modified to account for more complex variability in inter-mitotic times.

Mathematical models of the cell cycle often take a compartment-based approach, in which each compartment of the model represents a different cell cycle phase. In many of these models the dynamics are controlled by sub-cellular processes, such as the relative expression of cell cycle proteins (Bekkal Brikci et al 2008; Tyson and Novák 2001, 2022). Parametrising such models requires time-series data on these protein expression levels, which vary between cell lines and environmental conditions (such as oxygen availability).

Experimentalists may instead opt for simpler compartment-based models to describe the cell cycle. Here the rates of progress through each phase are assumed to be constant, and there are fewer model parameters. Several previous works look at population-balance, compartment-based ODE models (Celora et al 2022; Ubezio 2024), in which the proportion of cells in a given cell cycle phase settles to a steady state. Such ODE models consider the number of cells within a cell cycle phase, whereas structured PDE models view the cell cycle as a continuum, providing a more detailed description of the cell distribution. This raises questions about the extent to which the additional detail included in PDE models generates more insight into the system dynamics than simpler ODE models. Considering the discussion above about cell-cycle dependent anti-tumour treatment responses, such issues could be important when investigating optimal treatment strategies, although we leave explicit consideration of treatment to future work.

We choose two simple models of the cell cycle, a linear ODE model and a PDE model. We consider one of the simplest maturity-structured PDE models, as presented by Rubinow (1968), which introduces a time delay for progression of cells through each phase. The added complexity when compared to the linear ODE system acts as a proxy for more complicated biology. Thus, it is of interest to identify conditions under which the extra complexity included in the PDE model can be neglected and approximated by a simpler ODE model. We choose to leave consideration of how to fit these simple models to experimental data to future work, but note that this has been considered in the literature for several PDE and ODE models (Tyson et al 2012; Gabriel et al 2012; Ubezio 2024; Celora et al 2022).

The remainder of the paper is structured as follows. In Section 2, we introduce the general PDE model used by Rubinow (1968) and define simple functional forms to specify a continuously-structured model of a simplified cell cycle. Using the method of characteristics, we solve the PDE and use the solution to obtain expressions for quantities that could be measured by experimental data, such as the proportion of cells in each cell cycle phase, and the total population number. We also consider the behaviour of these quantities and find that we can average them over a single cell cycle to produce two constant quantities, namely the average population growth rate and average $\hbox {G}_2$ proportion, depending only on the PDE model parameters. In Section 3, we analyse a linear ODE system designed to model the same underlying process as the PDE model, and derive expressions for the steady-state growth rate and $\hbox {G}_2$ proportion in terms of the ODE parameters. In Section 4, we relate the two models by equating their analytic expressions for the growth rates and $\hbox {G}_2$ proportions. In this way, we obtain a mapping from a set of PDE parameters to a corresponding set of ODE model parameters. We explore the sensitivity of the ODE parameters to changes in the PDE parameters, and find regions of PDE parameter space in which the two sets of PDE parameters give rise to the same set of ODE parameters. We also consider how well the ODE model approximates the PDE model generated from these parameter mappings. We show further that fitting the ODE model to simulated PDE data without knowledge of the underlying parameters can lead to poor approximations.

In Section 5 we summarise our results and discuss ideas for further analysis of the two models, including parameter identifiability analysis, explicit modelling of treatment, and fitting to experimental data.

## Introducing the PDE Model

### Deriving the Model

Following Rubinow (1968), we introduce a PDE model of the cell cycle in which the continuous independent variable $\phi \in [0,1]$ represents a cell’s position in the cell cycle, where $\phi = 0$ corresponds to a cell at the beginning of the $\hbox {G}_1$ phase, and $\phi = 1$ corresponds to a cell at the end of the M phase. We neglect cell death under the assumption that the cells are in an optimal environment, with sufficient nutrient supply, no anti-tumour treatment applied and no growth-limiting spatial constraints. We assume that the dynamics of $p(\phi ,t)$, the density of cells with cell cycle position $\phi \in [0,1]$ at time $t>0$, can be described via the following PDE,

1 $$\begin{aligned} \frac{\partial p}{\partial t} + \frac{\partial }{\partial \phi }\left( v(\phi )p(\phi ,t)\right) = 0. \end{aligned}$$

In Equation (1), $v(\phi )$ denotes the rate at which a cell with phase $\phi$ progresses through the cell cycle, so that $v(\phi )p(\phi ,t)$ is the flux of cells with phase $\phi$ at time *t*. We prescribe the initial distribution of cells along the cell cycle continuum via the function

2 $$\begin{aligned} p(\phi ,0) = p_0(\phi ). \end{aligned}$$

We incorporate mitosis into the model by assuming that the flux of cells at $\phi = 0$ depends on the flux at $\phi = 1$ as follows:

3 $$\begin{aligned} v(0)p(0,t) = 2v(1)p(1,t). \end{aligned}$$

Equation (3) states that the flux of cells entering the cell cycle at the start of $\hbox {G}_1$ (where $\phi = 0$) is double the flux of cells exiting the cell cycle at the end of the *M*-phase (where $\phi = 1$). Thus, when cells with $\phi = 1$ divide, they produce two identical daughter cells with $\phi = 0$. We note that while we neglect cell death, Rubinow’s analysis accounts for cell death (Rubinow 1968). In this more general case, the following analysis holds with minimal changes to the qualitative behaviour of the results.

We note the equivalence of our PDE model for the cell cycle to timer, sizer and adder models for cell size control through sequential generations of cell proliferation (Facchetti et al 2017; Xia et al 2020; Rhind 2021; Rishal and Fainzilber 2019). Timer models have cells growing for a fixed amount of time before division. This can also be seen here via our definition of $v(\phi )$, where

4 $$\begin{aligned} \eta _1 = \int _0^1 \frac{1}{v(\phi )} d\phi \end{aligned}$$

is the fixed time taken for a cell to complete a full cell cycle. Alternatively, sizer models assume that each cell is aware of its own size, and will divide when its size reaches a target value. Our model is equivalent to this framework in that each cell has a $\phi$ value, and division occurs when $\phi = 1$. Similarly, in adder models the size of each cell must increase by a fixed increment from its birth size in order for proliferation to occur. The equivalence of this framework to our model is that all cells start with $\phi = 0$, and must increase to $\phi = 1$ for cell division to occur. Both the sizer and adder frameworks suggest that $\phi$ can be viewed as a proxy for cell size.

The PDE framework presented here assumes a fixed time interval between cell divisions (inter-mitotic time). We make this simplifying assumption to aid the analytic comparison of the PDE and ODE model later in this work. However, experimental work tracking the time taken for individual cells to divide has allowed for mathematical models to fit a probability distribution for the inter-mitotic time (Tyson et al 2012; Gabriel et al 2012; Stukalin et al 2013). The observed stochasticity in the inter-mitotic time has subsequently been used in several model frameworks, most notably in age-structured PDE models and stochastic birth-death process models (Stukalin et al 2013; Olofsson 2008; Olofsson and McDonald 2010; Maler and Lutscher 2010; Gabriel et al 2012; Xia et al 2020). The assumption that inter-mitotic times are correlated between mother-daughter and sister pairs of cells has also been considered mathematically (Lebowitz and Rubinow 1974; Webb 1986; Yan and Fu 2016), and a stochastic branching-process model has considered fitting to experimental data (Nordon et al 2011). Without loss of generality we fix $\phi _1 = 1/2$ to represent the boundary between the $\hbox {G}_1$ and the S/$\hbox {G}_2$/M phases.

In practice, $v = v(\phi )$ may vary continuously with $\phi$. However, we are unable to find experimental data that sheds light on possible biologically-realistic functional forms for $v(\phi )$ beyond the simplest assumption, which is that progression velocity is constant in each phase. In practice, these constant values can be found via FUCCI analysis (Sakaue-Sawano et al 2008; Jin et al 2021), which tags the cells with certain colours depending on their cell cycle phase. Thus, for simplicity and to aid comparison with a simple two-compartment ODE model (see Section 3), we assume that $v(\phi )$ is piecewise constant:

5 $$\begin{aligned} v(\phi ) = {\left\{ \begin{array}{ll} v, & \text {for } 0 \le \phi \le \frac{1}{2} \\ u, & \text {for } \frac{1}{2} < \phi \le 1. \end{array}\right. } \end{aligned}$$

With this definition of $v(\phi )$, we are, in effect, decomposing the cell cycle into two compartments, one for $\phi \in [0, 1/2]$ and another for $\phi \in [1/2, 1]$. We assume here that $\phi \in [0,1/2]$ corresponds to a cell in the $\hbox {G}_1$ phase, and that $\phi \in [1/2, 1]$ corresponds to a cell in the S/$\hbox {G}_2$/M phases (see Figure 1 for this simplified approach), which we will refer to as $\hbox {G}_2$ from now on. Thus, in the subsequent analysis, we decompose the cell cycle into two compartments, rather than three or four. We note here that the method proceeds identically in these more complex cases, and we discuss the differences in results in Section 2.3.1.

For simplicity, we also assume a uniform initial cell density in each phase, so that

6 $$\begin{aligned} p_0(\phi ) = {\left\{ \begin{array}{ll} p_0, & \text {for } 0 \le \phi \le \frac{1}{2} \\ q_0, & \text {for } \frac{1}{2} < \phi \le 1 \end{array}\right. } \end{aligned}$$

for some constants $p_0, q_0 \ge 0$.

With the boundary between phases fixed at $\phi _1 = 1/2$, we now use the functional form for $v = v(\phi )$ to determine cell cycle properties in terms of PDE model parameters. More specifically, the length of the cell cycle and the two phases are given by

7 $$\begin{aligned} \text {Cell cycle duration } = \eta _1 = \frac{1}{2v} + \frac{1}{2u}, \end{aligned}$$

8 $$\begin{aligned} \hbox { Duration of G}_1 = L_1 = \frac{1}{2v}, \end{aligned}$$

9 $$\begin{aligned} \hbox { Duration of G}_2 = L_2 = \frac{1}{2u}. \end{aligned}$$

These quantities will be useful later when analysing the solution to the PDE model.

### Solution for $p(\phi ,t)$

The piecewise-constant functional forms of $v(\phi )$ and $p_0(\phi )$ given by equations (5) and (6), respectively, allow us to implement the method of characteristics to solve the PDE (1).

For our specific choices of $v(\phi )$ and $p_0(\phi )$, the solution space partitions into three regions based on the lengths of each cell cycle phase.

We define $\eta _1 = L_1 + L_2$ to be the total cell cycle length, and *k*(*t*) as the number of full cell cycles completed since $t = 0$. More specifically,

10 $$\begin{aligned} k(t) = \lfloor \frac{t}{\eta _1}\rfloor . \end{aligned}$$

For clarity, we define $\tau = t + k(t)\eta _1$. Then the solution in each of these regions is given by:

- For $$0< \tau < \min (L_1, L_2)$$:
  11 $$\begin{aligned} p(\phi ,t) = {\left\{ \begin{array}{ll} 2^{k(t)+1}q_0 \frac{u}{v} & \text {for } 0< \phi< v \tau \\ 2^{k(t)} p_0 & \text {for } v \tau< \phi< \frac{1}{2} \\ 2^{k(t)}p_0\frac{v}{u} & \text {for } \frac{1}{2}< \phi< u \tau + \frac{1}{2} \\ 2^{k(t)}q_0 & \text {for } u \tau + \frac{1}{2}< \phi < 1. \end{array}\right. } \end{aligned}$$
- If $$v>u$$, for $$L_1< \tau < L_2$$:
  12 $$\begin{aligned} p(\phi ,t) = {\left\{ \begin{array}{ll} 2^{k(t)+1}q_0 \frac{u}{v} & \text {for } 0< \phi< \frac{1}{2} \\ 2^{k(t)+1} q_0 & \text {for } \frac{1}{2}< \phi< u \cdot (\tau + \frac{1}{2u} - \frac{1}{2v})\\ 2^{k(t)}p_0\frac{v}{u} & \text {for } u \cdot (\tau + \frac{1}{2u} - \frac{1}{2v})< \phi< u \cdot (\tau + \frac{1}{2u}) \\ 2^{k(t)}q_0 & \text {for } u \cdot (\tau + \frac{1}{2u})< \phi < 1. \end{array}\right. } \end{aligned}$$
- If $$u>v$$, for $$L_2< \tau < L_1$$:
  13 $$\begin{aligned} p(\phi ,t) = {\left\{ \begin{array}{ll} 2^{k(t)+1}p_0 & \text {for } 0< \phi< v \cdot \left( \tau - \frac{1}{2u}\right) \\ 2^{k(t)+1} q_0\frac{u}{v} & \text {for } v \cdot \left( \tau - \frac{1}{2u}\right)< \phi< v \tau \\ 2^{k(t)}p_0 & \text {for } v \tau< \phi< \frac{1}{2} \\ 2^{k(t)}p_0 \frac{v}{u}& \text {for } \frac{1}{2}< \phi < 1. \end{array}\right. } \end{aligned}$$
- For $$\max (L_1,L_2)< \tau < \eta _1$$:
  14 $$\begin{aligned} p(\phi ,t) = {\left\{ \begin{array}{ll} 2^{k(t)+1}p_0 & \text {for } 0< \phi< v \tau - \frac{v}{2u} \\ 2^{k(t)+1} q_0\frac{u}{v} & \text {for } v \tau - \frac{v}{2u}< \phi< \frac{1}{2}\\ 2^{k(t)+1}q_0 & \text {for } \frac{1}{2}< \phi< u \cdot (\tau + \frac{1}{2u} - \frac{1}{2v}) \\ 2^{k(t)}p_0 \frac{v}{u}& \text {for } u \cdot (\tau + \frac{1}{2u} - \frac{1}{2v})< \phi < 1. \end{array}\right. } \end{aligned}$$

**Fig. 2:**
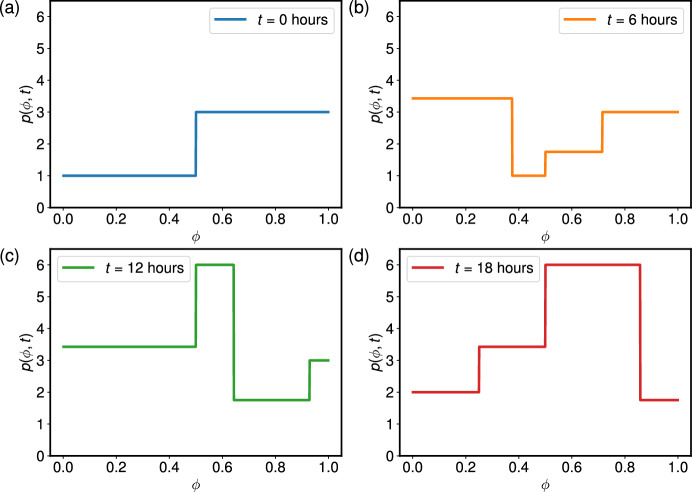
Solutions for $p(\phi ,t)$ to highlight three of the four solutions (11)-(14), where $v = 1/16$, $u = 1/28$, $p_0 = 1$, $q_0 = 3$. We plot the density profile $p(\phi ,t)$ at four times: (a) $t = 0$ hours, given by equation (11), (b) $t=6$ hours, given by equation (11), (c) $t = 12$ hours, given by equation (12), and (d) $t = 18$ hours, given by equation (14).

Therefore, splitting the solution up into three cases based on the value of *t* allows us to find the solution for any $\phi \in (0,1)$ with this given $t>0$. For a fixed $t>0$, we see that the solution splits the $\phi$-domain into four sections, with a constant cell density in each region. This is illustrated in Figure 2, where we plot the cell density $p(\phi ,t)$ as $\phi$ varies for different values of $t\ge 0$. We choose the three values of *t* to be within a single cell cycle, and to cover all three of the cases from equations (11)-(14) that occur for a single set of parameters $(v,u,p_0, q_0)$. In each case where $t \ne 0$, we see four regions where the solution is constant.

### Phase Counts and Proportions

We denote by $p_{G_1}(t)$ and $p_{G_2}(t)$ the number of cells in each phase, and note that

15 $$\begin{aligned} p_{G_1}(t) = \int _0^{\frac{1}{2}} p(\phi ,t) d\phi \hspace{0.5cm} \text { and } \hspace{0.5cm} p_{G_2}(t) =\int _{\frac{1}{2}}^1 p(\phi ,t)d\phi , \end{aligned}$$

respectively. Using these expressions we can define the cell phase proportions via

16 $$\begin{aligned} \pi _{1}(t) = \frac{p_{G_1}(t)}{N(t)} \hspace{0.5cm} \text { and } \hspace{0.5cm} \pi _{2}(t) = \frac{p_{G_2}(t)}{N(t)}, \end{aligned}$$

where $$N(t) = p_{G_1}(t) + p_{G_2}(t)$$.

In each of the four cases (11)-(14) (where only three will be present for a given parameter set, $(v,u,p_0,q_0)$), the solution is piecewise constant in each of four sub-intervals of the $\phi$ domain. Therefore, the integrals are easy to calculate as the sum of four constants, and the results for $\pi _{G_2}(t)$ and *N*(*t*) in each case are as follows:

- For $$0< \tau < \min (L_1, L_2)$$:
  17 $$\begin{aligned} N(t) = 2^{k(t)} \left[ \frac{1}{2}(p_0+q_0) + uq_0(t-k(t)\eta _1)\right] , \end{aligned}$$
  18 $$\begin{aligned} \pi _{G_2}(t) = \frac{\frac{1}{2}q_0 + (p_0v - q_0u)\tau }{\frac{1}{2}(p_0+q_0) + uq_0(t-k(t)\eta _1)}. \end{aligned}$$
- If $$v>u$$, for $$L_1< \tau < L_2$$:
  19 $$\begin{aligned} N(t) = 2^{k(t)} \left[ \frac{1}{2}\left( p_0 + q_0\right) + uq_0\left( \tau \right) \right] , \end{aligned}$$
  20 $$\begin{aligned} \pi _{G_2}(t) = \frac{\frac{1}{2}(p_0 + q_0) - \frac{u}{v}q_0 + uq_0\tau }{\frac{1}{2}\left( p_0 + q_0\right) + uq_0\tau }. \end{aligned}$$
- If $$u>v$$, for $$L_2< \tau < L_1$$:
  21 $$\begin{aligned} N(t) = 2^{k(t)}\left[ q_0 + \left( \frac{1}{2} - \frac{v}{2u}\right) p_0 + vp_0\tau \right] , \end{aligned}$$
  22 $$\begin{aligned} \pi _{G_2}(t) = \frac{\frac{v}{2u}p_0}{q_0 + \left( \frac{1}{2} - \frac{v}{2u}\right) p_0 + vp_0\tau }. \end{aligned}$$
- For $$\max (L_1,L_2)< \tau < \eta _1$$:
  23 $$\begin{aligned} N(t) = 2^{k(t)} \left[ q_0 + \left( \frac{1}{2} - \frac{v}{2u}\right) p_0+ vp_0\tau \right] , \end{aligned}$$
  24 $$\begin{aligned} \pi _{G_2}(t) = \frac{\left( \frac{v}{2u} + \frac{1}{2}\right) p_0 - \frac{u}{v}q_0 + (2uq_0 - vp_0)\tau }{q_0 + \left( \frac{1}{2} - \frac{v}{2u}\right) p_0+ vp_0\tau }. \end{aligned}$$

#### Periodicity and average values

Recalling the definition of *k*(*t*):

25 $$\begin{aligned} k(t) = \lfloor \frac{t}{\frac{1}{2v}+\frac{1}{2u}} \rfloor = \lfloor \frac{t}{\eta _1} \rfloor , \end{aligned}$$

we see that $k(t+\eta _1) = k(t)+1$. Therefore, for each $t>0$,

26 $$\begin{aligned} \begin{aligned} (t + \eta _1) - k(t+\eta _1)\eta _1&= (t + \eta _1) - (k(t) + 1)\eta _1 \\&= t - k(t)\eta _1 \\&= \tau . \end{aligned} \end{aligned}$$

Using (26) in equations (18), (20), (22), (24), we deduce that $\pi _{G_2}(t)$ is periodic in *t*, with period equal to the cell cycle length $\eta _1$. We note that periodicity persists independent of the functional form of $v(\phi )$. Thus, one could use a different $v(\phi )$ in equation (5) and obtain qualitatively similar results. Furthermore, the addition of a death term, as in Rubinow (1968), would preserve periodicity, but increase the complexity of the analysis.

We now exploit the periodicity of $\pi _{G_2}(t)$ to determine its mean value over a cell cycle. This mean value, ${\bar{\pi }}_{2}$, is given by

27 $$\begin{aligned} {\bar{\pi }}_{2} = \frac{1}{\eta _1} \int _0^{\eta _1} \pi _{G_2}(t) dt. \end{aligned}$$

By referring to equations (18)-(24), we note that the expressions for $\pi _{G_2}(t)$ can be written in the general form

28 $$\begin{aligned} \pi (t) = \frac{a+b\tau }{c + d \tau }, \end{aligned}$$

where *a*, *b*, *c* and *d* are known constants. As we need only consider $t \in [0, \eta _1)$ for the mean value by periodicity, we put $k(t) = 0$ in these calculations.

To simplify the calculation, we reframe the initial conditions (6) as $q_0 = sp_0$, for some positive constant *s*. After some algebra, we find that when $L_1>L_2$, the average proportion of cells in the G$_2$ phase, ${\bar{\pi }}_{2}$, is given by

29 $$\begin{aligned} 2s^2u^2v^2\eta _1{\bar{\pi }}_{2} =&\left[ 4u^3s^4 + 4(u^3 - u^2v)s^3 + (uv^2 - 2u^2v)s^2\right] \log \left( s+1-\frac{v}{2u}\right) \nonumber \\&+ \left[ -4u^3s^4 + 4(u^2v - u^3)s^3\right] \log (1+s) \nonumber \\&+ \left[ 2(u^2v-uv^2)s^2 + (v^3-uv^2)s+v^3\right] \log (1+s) \nonumber \\&+ \left[ uv^2s^2 + (uv^2-v^3)s - v^3\right] \log \left( s + \frac{1}{2}\right) \nonumber \\&+ \left[ 2uv^2s^2 + (uv^2-v^3)s - v^3\right] \log (2) + 2u^2vs^3 -2uv^2s^2 + v^3s, \end{aligned}$$

whilst when $$L_2>L_1$$,

30 $$\begin{aligned} \begin{aligned} 2s^2u^2v^2\eta _1 {\bar{\pi }}_{2} =&\left[ 2(u^2v+uv^2)s^2 + (uv^2-v^3)s - v^3\right] \log \left( s+1 + \frac{u}{v}\right) \\&+\left[ -4u^3s^4+4(u^2v-u^3)s^3\right] \log (1+s) \\&+ \left[ 2(u^2v-uv^2)s^2 + (v^3-uv^2)s+v^3\right] \log (1+s) \\&+\left[ 4u^3s^4 + 4(u^3-u^2v)s^3 - 4u^2vs^2\right] \log \left( s + \frac{1}{2}\right) \\&-2u^2vs^2\log (2) + 2u^3s^3 + (uv^2 - 3u^2v)s^2 + uv^2s. \end{aligned} \end{aligned}$$

Furthermore, we can use the expressions (17), (19), (21) and (23) for the total population numbers to find the growth rate of the population. To illustrate the process, we use the case in which the G$_2$ phase is longer than the G$_1$ phase (corresponding to $u<v$), and note that the analysis can be repeated in the same way for the reverse case. If we denote the total cell population at time $t>0$ by *N*(*t*), we find from equations (17) - (23) that

31 $$\begin{aligned} N(t) = {\left\{ \begin{array}{ll} 2^{k(t)} \left[ \frac{1}{2}(1+s) + su\tau \right] p_0 & \text {for } 0< \tau< \frac{1}{2u}, \\ 2^{k(t)} \left[ s + \left( \frac{1}{2} - \frac{v}{2u}\right) + v \cdot (t-k(t)\eta _1)\right] p_0 & \text {for } \frac{1}{2u}< \tau < \eta _1, \end{array}\right. } \end{aligned}$$

where *k*(*t*) is the positive integer defined by $k(t) = \lfloor t/\eta _1 \rfloor$. For any integer $K\ge 0$, consider $t \in (K\eta _1, (K+1)\eta _1)$ so that $k(t) = K$ remains constant in this interval. Then we can differentiate (31) with respect to *t* to obtain

32 $$\begin{aligned} N'(t) = {\left\{ \begin{array}{ll} 2^Kusp_0 & \text {for } 0< t - K\eta _1< \frac{1}{2u}, \\ 2^Kvp_0 & \text {for } \frac{1}{2u}< t - K\eta _1 < \eta _1, \end{array}\right. } \end{aligned}$$

where $N'(t)$ denotes the derivative with respect to time of the total population number.

Therefore, for any fixed integer $$K\ge 0$$ the population growth rate, $$\beta (t)$$, at time $$t >0 $$ is given by

33 $$\begin{aligned} \beta (t) = \frac{N'(t)}{N(t)} = {\left\{ \begin{array}{ll} \frac{su}{\frac{1}{2}(1+s) + su(t-K\eta _1)} & \text {for } 0< t - K\eta _1<\frac{1}{2u}, \\ \frac{v}{s + \left( \frac{1}{2} - \frac{v}{2u}\right) + v \cdot (t - K\eta _1)} & \text {for } \frac{1}{2u}< t -K\eta _1 < \eta _1. \end{array}\right. } \end{aligned}$$

We note that the growth rate is time-periodic with period $\eta _1$, and so we can calculate the mean growth rate ${\bar{\beta }}$ over a single period $t \in [0, \eta _1]$, where $K = 0$, as follows

34 $$\begin{aligned} \begin{aligned} {\bar{\beta }}&= \frac{1}{\eta _1}\int _0^{\eta _1} \beta (t) dt \\&= \frac{1}{\eta _1}\int _0^{\frac{1}{2u}} \frac{su}{\frac{1}{2}(1+s) + sut} d{\tilde{\eta }} +\frac{1}{\eta _1} \int _{\frac{1}{2u}}^{\eta _1}\frac{v}{s + \frac{1}{2} - \frac{v}{2u} + vt} dt \\&= \frac{\log (2)}{\eta _1}. \end{aligned} \end{aligned}$$

Note that this is the growth rate that we estimate if we assumed exponential growth for the population.

We pause here to consider the impact of splitting the $\phi$ axis into more compartments in order to represent each cell cycle phase individually. We could extend our piecewise constant functional forms for $p_0(\phi )$ and $v(\phi )$ to account for these extra compartments and repeat the above analysis. This would not affect the periodicity of the resulting solutions. However, increasing the number of compartments increases the number of cases for consideration. For example, with two compartments there are 4 different cases based on the value of *t* and the relative lengths of the compartment, and each solution comprises four piecewise components. In the three compartment case, we must consider the relative lengths of the three phases, and also their pairwise sums. Thus, if *a*, *b*, *c* represent the lengths of the three phases, respectively, we would need to know how *a*, *b*, *c*, $a+b$, $a+c$, and $b+c$ are all ordered. There are 12 different arrangements of these lengths, each of which would require a piecewise solution with 6 components for $p(\phi ,t)$. Of these 12 solutions, 7 would be needed to span a single period of *t*, namely $[0, \eta _1]$. Thus determining the phase proportions would be more cumbersome to calculate. We expect the number of cases to consider to increase significantly if four compartments were considered.

**Fig. 3:**
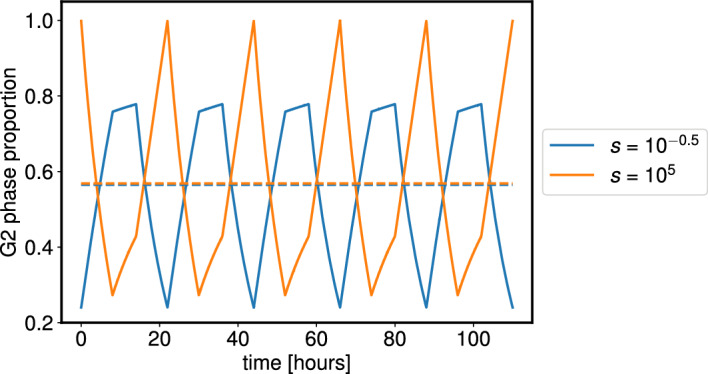
Cell cycle phase proportion dynamics for the G$_2$ phase over the time-span of 5 complete 22-hour cell cycles show sharp periodic oscillations. Here  $v =1/28$, $u = 1/16$, and we use two different initial conditions, with $s = 10^{-0.5}$ (blue curves) and $s = 10^5$ (orange curves). The corresponding curves for the phase proportions of the G$_1$ phase are omitted here, but can be found using $\pi _{G_2}(t) = 1 - \pi _{G_1}(t)$. The dashed lines represent the mean value of the oscillatory curve of the corresponding colour over a single period, $\eta _1 = 22$ hours. The two values of *s* were chosen such that the mean values would be similar.

**Fig. 4:**
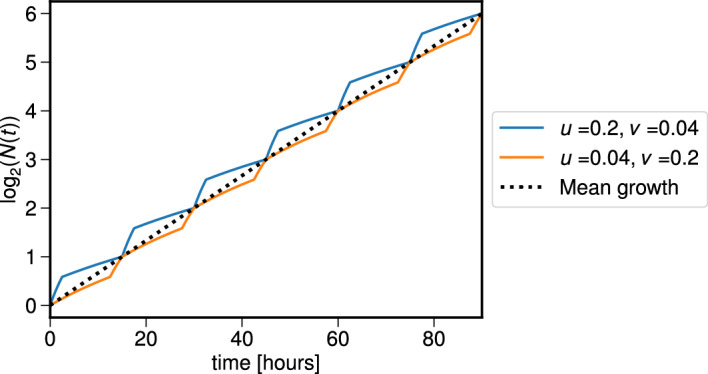
The relative values of the two phase velocities drive differences in population growth in each period. Here we fix $\eta _1 = 15$ hours, and plot the log-scaled population *N*(*t*) for two pairs of (*v*, *u*) values. The blue curve shows the growth for $(v,u) = (0.04, 0.2)$, whilst the orange curve uses $(v,u) = (0.2, 0.04)$. The population size found using $\exp ({\bar{\beta }}t)$, where ${\bar{\beta }}$ is the mean value of the growth rate (see equation (34)) is given by the black dashed line. We fix $s = 1$ in each case.

We use the expressions calculated above in Figure 3 to plot the cell cycle phase proportions for fixed values of *v* and *u* (with $v<u$) and two choices of initial conditions. The simulations show how the dynamics change as the proportions of cells initially in the G$_1$ and G$_2$ phases vary. The mean values of the oscillations over a single period, calculated using equation (29), are very similar in the two cases.

We note from equations (29) and (30) that $\pi _{G_1}(t)$ and $\pi _{G_2}(t)$ are independent of the parameter $p_0$. This is because the initial proportions $\pi _{G_1}(t = 0)$ and $\pi _{G_2}(t = 0)$ depend on *s* and not $p_0$.

Figure 4 shows how the total cell population *N*(*t*) evolves for different values of (*v*, *u*) when the cell cycle length is fixed at $\eta _1 = 1/2v + 1/2u = 15$ hours. For each (*v*, *u*) pair, the total cell population takes the same value of *N*(*t*) whenever $t = m \eta _1$ for some integer $m>0$, but each pair reaches this value in different ways for $t \in [m\eta _1, (m+1)\eta _1]$. We see that when the G$_1$ velocity is smaller than that of G$_2$, the population is larger than the population resulting from the “average" growth value in equation (34), whenever $t \ne m\eta _1$ (and conversely when the two phase velocities are swapped).

In conclusion, for a fixed cell cycle length $\eta _1$, different combinations of the cell cycle progression velocities (*v*, *u*) that satisfy $\eta _1 = 1/2v + 1/2u$ can generate identical total cell population values when measured at fixed time intervals, $t = m\eta _1$, for integers $m>1$. However, the growth dynamics of the populations at intermediate times may differ.

In summation, we have derived the general solution for a simple, continuously-structured PDE model of the cell cycle. We have used the resulting analytical expressions to derive expressions for the population growth rate, and the proportion of cells in each cell cycle phase. These expressions are periodic, and so can be utilised to derive analytical expressions for the mean growth behaviour of the PDE system.

In the next section, we present a simpler ODE system, and then consider how we can relate both models.

## Introducing the ODE Model

### Simple ODE Model

In this section we analyse a linear ODE model corresponding to the PDE model. As for the structured model, we decompose the cell cycle into two phases which we term $G_1(t)$ and $G_2(t)$ ($\equiv$ S $+$ G$_2$ $+$ M), and consider the simplest representation of cells moving between the two phases at some given rates. Assuming that there is no cell death in the system, we propose the following pair of ODEs to describe the population growth

35 $$\begin{aligned} \frac{dG_1}{dt}= &  2k_2 G_2 - k_1 G_1, \nonumber \\ \frac{dG_2}{dt}= &  k_1 G_1 - k_2 G_2, \end{aligned}$$

with

36 $$\begin{aligned} G_1(0) = G_1^0, \hspace{0.5cm} G_2(0) = G_2^0, \end{aligned}$$

where $G_1^0$ and $G_2^0$ are non-negative constants. In equations (35), $k_1$ and $k_2$ denote the rates at which cells in the G$_1$ and G$_2$ compartments, respectively, move into the next compartment. The factor of two in the ODE for $G_1(t)$ ensures that when cell division occurs at the end of the G$_2$ phase, two daughter cells enter the G$_1$ population. As with the PDE model, we wish to know how the total population $N(t) = G_1(t) + G_2(t)$ and the phase proportions $g_1(t) = G_1(t)/N(t)$ (and $g_2(t) = G_2(t)/N(t)$) evolve over time. Using equations (35), it is straightforward to show that

37 $$\begin{aligned} \frac{1}{N} \frac{dN}{dt} = k_2 g_2, \end{aligned}$$

38 $$\begin{aligned} \frac{dg_2}{dt} = k_1 - (k_1+k_2)g_2 - k_2g_2^2. \end{aligned}$$

In order to find a relationship between the ODE model parameters $(k_1, k_2)$ and the PDE model parameters (*v*, *u*, *s*), we consider the behaviour of the ODE model at large times.

By setting the time derivative equal to zero in equation (38), we find that the stable steady state G$_2$ cell cycle proportion, ${\tilde{g}}_2$, is

39 $$\begin{aligned} {\tilde{g}}_2 = \frac{-k_2 - k_1 + \sqrt{k_2^2 + 6k_1k_2 + k_1^2}}{2k_2} \in [0, 1]. \end{aligned}$$

Recalling that the population growth rate is given by $N'(t)/N(t)$, we use (39) in (37) to find that the long-term growth rate of the population is given by

40 $$\begin{aligned} \lambda ^+ = \frac{-(k_1 + k_2) + \sqrt{k_2^2 + 6k_1k_2 + k_1^2}}{2}. \end{aligned}$$

As with the PDE model, this analysis could be extended to account for four cell cycle phases without affecting the qualitative results. Coupled linear ODEs would describe the dynamics of each phase and the cell population would undergo asynchronous exponential growth (Gyllenberg and Webb 1992), i.e. the total number of cells grows exponentially, but the proportion of cells in each phase settles to a steady-state value.

Equations (39) and (40) define two measurable model quantities $\lambda ^+$ and ${\tilde{g}}_2$ in terms of the ODE model parameters. We can use these expressions to estimate $k_1$ and $k_2$ given measured values of $\lambda ^+$ and ${\tilde{g}}_2$, via the expressions

41 $$\begin{aligned} k_2(\lambda ^+, {\tilde{g}}_2) = \frac{\lambda ^+}{{\tilde{g}}_2}, \end{aligned}$$

and

42 $$\begin{aligned} k_1(\lambda ^+, {\tilde{g}}_2) = \frac{ {\tilde{g}}_2 + 2\lambda ^+}{2\left( 1 - {\tilde{g}}_2 \right) }. \end{aligned}$$

The quantities $\lambda ^+$ and ${\tilde{g}}_2$ align closely with the average population growth rate and cell phase proportion calculated for the PDE model in Section 2, respectively. The parameter *s* from the PDE model has an analogue in the ODE model, found by noting $s = G_2(0)/G_1(0)$. However, the expressions for ${\tilde{g}}_2$ and $\lambda ^+$ are independent of the initial conditions. Instead, the initial proportion of cells in each phase will dictate how long the system takes to converge to the steady state phase proportion and growth rate (given by equations (39) and (40)). Whilst not shown here, the larger the difference between the initial and equilibrium phase proportions, the longer it takes for the system to evolve to its equilibrium value. In the next section, we will use the expressions to compare the ODE and PDE models.

## PDE-ODE Comparison

One way to compare the two models is to integrate the PDE (1) over the phase intervals, $\phi \in [0, 1/2]$ and $\phi \in [1/2, 1]$. This method is considered by Sundareshan and Fundakowski (1984) under the assumption that the cell density is approximately constant over each sub-interval of the cell cycle. This assumption is not valid for our two compartment model where the cell density may vary greatly within a cell cycle phase due to the sharp jump in population numbers caused by cell division. However, armed with expressions for the long-term population growth rate and steady-state cell phase proportions for the ODE model, and the average population growth rate and average cell phase proportion over a single cell cycle for the PDE model, we seek to relate the ODE model parameters $(k_1, k_2)$ to those of the PDE, (*v*, *u*, *s*). We will then use these relationships to compare the models’ behaviour.

### Relating Parameters

We compare the ODE and PDE models by equating the mean values of the cell proportions (without loss of generality we can focus on just one proportion) and overall growth rate over a cell cycle period. The quantities of interest from the ODE model are $\lambda ^+(k_1,k_2)$ and ${\tilde{g}}_2(k_1,k_2)$, where

43 $$\begin{aligned} \lambda ^+(k_1,k_2) = \frac{-(k_2 + k_1) + \sqrt{k_1^2 + 6k_1k_2 + k_2^2}}{2}, \end{aligned}$$

44 $$\begin{aligned} {\tilde{g}}_2(k_1, k_2) = \frac{-(k_2 + k_1) + \sqrt{k_1^2 + 6k_1k_2 + k_2^2}}{2k_2}. \end{aligned}$$

The corresponding quantities from the PDE model are ${\bar{\beta }}(v,u)$ and ${\bar{\pi }}_{2}(v,u,s)$, where

45 $$\begin{aligned} {\bar{\beta }}(v,u) = \frac{2uv\log {2}}{u+v}, \end{aligned}$$

and ${\bar{\pi }}_{2}(v,u,s)$ is defined by equations (29) (when $v<u$) and (30) (when $v>u$).

We want to find the values $(k_1, k_2)$ for which we have

46 $$\begin{aligned} {\bar{\beta }}(v,u) = \lambda ^+(k_1,k_2), \end{aligned}$$

and

47 $$\begin{aligned} {\bar{\pi }}_{2}(v, u, s) = {\tilde{g}}_2(k_1, k_2), \end{aligned}$$

so that we can find a corresponding set of ODE parameters for a given set of PDE parameters. We will refer to the ODE model with parameters derived from the PDE model using equations (46) and (47) as the “corresponding" ODE model.

**Fig. 5:**
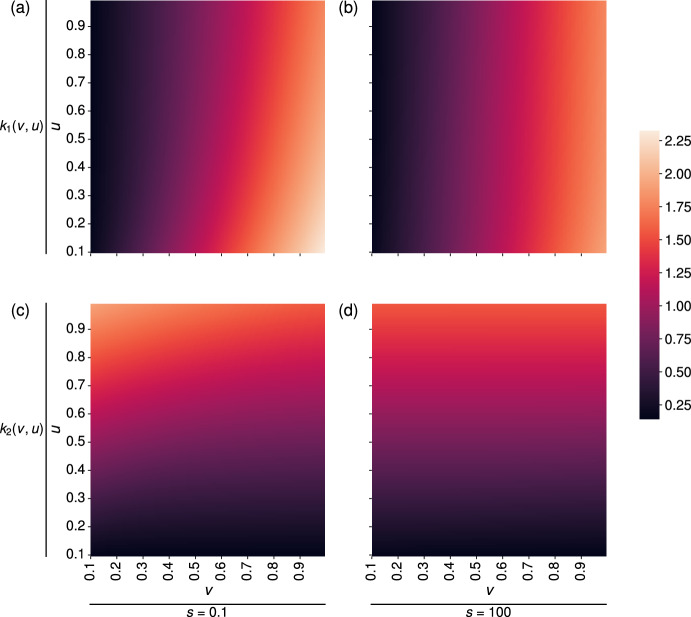
When matching coefficients between models, the value of $$k_2$$ seems to be most sensitive to changes in the G$$_2$$ phase velocity, *u*, whilst $$k_1$$ appears to be most sensitive to changes in the $$G_1$$ phase velocity, *v*. Here, we display how the estimated values of $$k_1$$ and $$k_2$$ in the ODE model change as we vary the parameters *v* and *u* in the PDE model, when $$s =0.01 $$ (panels (a) and (c)), and $$s = 100$$ (panels (b) and (d)).

To get a sense of how $k_1$ and $k_2$ depend on the PDE parameters, we start by fixing *s* and using equations (46) and (47) to determine $k_1 = k_1(v,u)$ and $k_2 = k_2(v,u)$. Figure 5 shows how $k_1$ and $k_2$ vary for two fixed values of *s*. In each row of plots, we set the minimum and maximum of the colour-bar to be the overall minimum and maximum across both *s*-cases to more easily compare the plots. In both cases, the range of values of $k_1$ and $k_2$ are similar, with the smaller value of *s* able to reach the highest values of $k_1$ and $k_2$. In general, $k_1$ increases with *v*, and $k_2$ increases with *u*. We see slight decreases in $k_1$ by increasing *u* for a fixed *v*, and vice-versa for $k_2$.

**Fig. 6:**
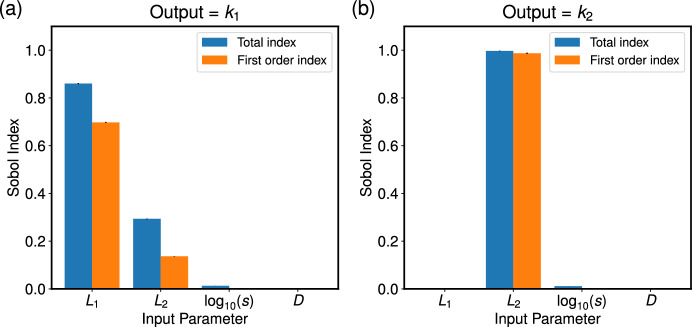
Sobol indices for the ODE parameters $k_1$ (panel (a)) and $k_2$ (panel (b)) as functions of the PDE parameters, (*v*, *u*, *s*), found using equations (46) and (47), show that variance in $k_1$ and $k_2$ is largely controlled by $L_1$, $L_2$ and their pairwise interaction, and solely $L_2$, respectively. A dummy parameter, *D*, is included as a negative control. We allow the duration of the G$_1$ and G$_2$ phases ($L_1$ and $L_2$, respectively) to vary from 1 hour to 22 hours, and $\log _{10}(s)$ to vary between $-5$ and 5. The vertical black lines on each bar represent the 95$\%$ confidence interval for each index.

In order to investigate the variance in the matched ODE parameters with respect to changes to each PDE input parameter, we perform a global sensitivity analysis on the parameters $k_1 = k_1(v,u,s)$ and $k_2 = k_2(v,u,s)$. Using the Python package SALib (Herman and Usher 2017; Iwanaga et al 2022), we calculate the Sobol indices for each input parameter *u*, *v* and *s* and present the results in Figure 6. Briefly, the Sobol indices describe how the variance in model outputs can be attributed to each model input. Here, our model inputs are $L_1$, $L_2$ and *s*, and we calculate the Sobol indices for two model outputs, the ODE parameters $k_1$ and $k_2$. The first order Sobol indices (orange bars in Figure 6) show the contribution to the output variance when each input parameter is varied alone. The total Sobol indices (blue bars in Figure 6) denote the contribution to the output variance from varying each input parameter, including interactions between different input parameters. We also include a dummy variable, *D*, which acts as a “negative control" (Marino et al 2008). As expected, the Sobol indices of the dummy parameter are zero (see Figure 6). Thus, the Sobol indices and dummy variable provide a measure of significant values for model parameters.

Using the fact that $(2v)^{-1}$ and $(2u)^{-1}$ represent the time taken for a cell to progress through the G$_1$ and G$_2$ phases, respectively, we choose a range of values for *u* and *v* so that our samples are consistent with typical cell timescales for the G$_1$ and G$_2$ phases. In both cases, we see that the value of $\log _{10}(s)$ has little influence on the ODE parameters.

In Figure 6(a), both $L_1$ and $L_2$ contribute to the variance of $k_1$, with $L_1$ having a higher total and first order Sobol indices than $L_2$. The interaction between $L_1$ and $L_2$ was the only second-order effect needed to account for the discrepancy between the total and first order indices of both $L_1$ and $L_2$, with a second order Sobol index of approximately 0.15 (the second-order indices formed by pairwise interactions of the input parameters are not shown here as the majority are negligible).

Figure 6(b) demonstrates that the length of the G$_2$ phase (defined by $L_2 = 1/(2u)$) contributes almost all of the variance in the value of $k_2$, with no significant contribution from any second-order interactions. These results are consistent with those in panels (a) and (b) of Figure 5, where $k_1$ varies with *u*, but to a lesser extent than with *v*.

### Multi-Valued Functions

Three degrees of freedom in the PDE model (namely *v*, *u* and *s*) determine the quantities ${\bar{\pi }}_{2}$ and ${\bar{\beta }}$ and, hence, the rate constants, $k_1$ and $k_2$, in the ODE model. Therefore, we might expect that multiple sets of PDE parameters are able to generate a single ODE parameter set. To investigate this, we fix a pair of ODE parameters $(k_1, k_2)$, and solve the inverse problem to find PDE parameter sets (*v*, *u*, *s*) for which the ODE approximates the average behaviour of the PDE.

In deriving the expressions for the growth rates and G$_2$ proportions, we found that the growth rate is a function of $k_1$ and $k_2$ if taking the ODE approach (equation (40)), and a function of only *u* and *v* if taking the PDE approach (equation (34)). Therefore, fixing $k_1$ and $k_2$ fixes ${\bar{\beta }}$, which then produces a fixed relationship between *u* and *v*, namely

48 $$\begin{aligned} \lambda ^{+}(k_1, k_2) = {\bar{\beta }}(v,u) = \frac{2uv\log {2}}{u+v}. \end{aligned}$$

Hence we see that fixing $k_1$ and $k_2$ constrains the values of *u* and *v* to lie on a single-valued curve in (*v*, *u*)-space, defined by (48).

**Fig. 7:**
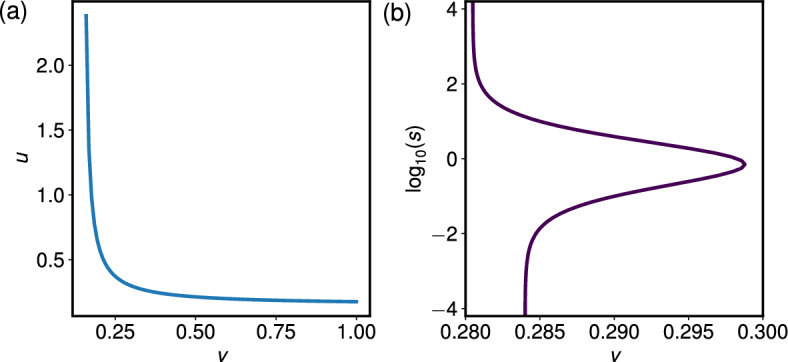
Fixing the average growth rate of the population restricts (*v*, *u*) to a simple curve, whilst fixing the average G$_2$ proportion allows for cases in which two distinct initial phase proportions are possible for a single (*v*, *u*) pair. (a) Contour in (*v*, *u*)-space that satisfies ${\bar{\beta }}(v,u) = \lambda ^+(k_1 = 0.5, k_2 = 0.5)$, found using (43) and (48). (b) Contour in $(v, \log _{10}(s))$-space that satisfies ${\bar{\pi }}_2(v,u,s) = {\tilde{g}}_2(k_1 = 0.5,k_2 =0.5)$, where $u = u(v)$ is defined by fixing ${\bar{\beta }}(v,u) = \lambda ^+(k_1 = 0.5, k_2 = 0.5)$.

We present an example of this in Figure 7(a), where we fix ${\bar{\beta }}(v,u) = \lambda ^+(k_1 = 0.5, k_2 = 0.5)$ and find the corresponding (*v*, *u*) pairs using (48). With $u = u(v)$ as taken from Figure 7(a), Figure 7(b) presents the $(\log _{10}(s),v)$ pairs that are required to satisfy ${\bar{\pi }}_2(v,u,s) = {\tilde{g}}_2(k_1 = 0.5,k_2 = 0.5)$. We see there are regions in (*v*, *u*) parameter space such that different initial conditions map to the same point in $(k_1,k_2)$ parameter space. More specifically, for some progression velocity pairs (*v*, *u*), we find two distinct values of the initial cell ratio parameter *s*, $(s_1,s_2)$, such that $k_1(v,u,s_1) = k_1(v,u,s_2)$ and $k_2(v,u,s_1) = k_2(v,u,s_2)$. These distinct values of *s* alter the initial distribution of the cells over the cell cycle in the PDE model, and hence alter the time-evolution of the solution $p(\phi ,t)$ together with producing different amplitudes of oscillation for $\pi _{2}(t)$.

In summary, we find that there are regions of parameter space such that a single pair of ODE parameters $(k_1, k_2)$ can be obtained via two distinct sets of PDE parameters, $(v,u,s_1)$ and $(v, u, s_2)$, where the initial condition varies, i.e. $s_1 \ne s_2$. By design, the long-time ODE behaviour matches the average PDE behaviour. This suggests that only knowing the average behaviour cannot uniquely specify the PDE behaviour and, in particular, the initial cell distribution. We conclude that, when applying phase-dependent treatments, knowing the initial conditions of the PDE model is vital, and we will demonstrate this in Section 4.4.

### Realistic Values of Cell Cycle Progression

Having shown that, by suitable choice of the initial conditions (with control parameter *s*), multiple sets of parameters (*v*, *u*, *s*) can give rise to the same values of $(k_1,k_2)$, we now focus on biologically realistic values of *v* and *u*.

Cell cycle length (and individual cell cycle phase length) varies between different cells lines, with some variation present between cells of the same cell line (Weber et al 2014). Experimental data from different cell lines suggest that the cell cycle length of human cells can range between 18-24 hours, with phase lengths also variable (Weber et al 2014; Araujo et al 2016; Cotton et al 2024; Eidukevicius et al 2005; Cooper 2000). In line with this range of estimates, we fix $\eta _1 = 22$ hours. Then ${\bar{\beta }} = \log (2)/22$, and $1/v + 1/u = 44$ by equation (48), thus restricting *u* and *v* to the simple curve in Figure 7(a). Estimates for the G$_1$ phase length range from 4-6 hours in human HT1080 fibrosarcoma cells (Marcus et al 2015) to around 8 hours in primary human intestinal epithelial cells (Cotton et al 2024) to around 11 hours for rapidly proliferating cells (Cooper 2000). From this, we choose $v = 1/16$ and $u = 1/28$ to represent the G$_1$ phase being 8 hours long, and the G$_2$ phase being 14 hours long, respectively.

**Fig. 8:**
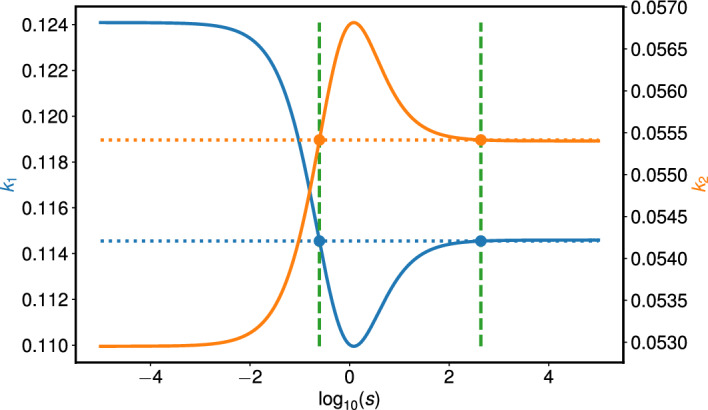
An example highlighting that there can be two values of *s* that give rise to a single $(k_1, k_2)$ pair, as illustrated by the two green dashed lines. We plot the ODE parameters, $k_1$ (blue) and $k_2$ (orange), as *s* varies when $u = 1/28$ and $v = 1/16$. The blue dots correspond to $k_1 = 0.11455$, and the orange dots are $k_2 = 0.05541$ (see Figure 9).

Figure 8 shows how $k_1$ and $k_2$ change as *s* varies using (42) and (41). We see that as *s* varies within the range $10^{-5}< \log _{10}(s) < 10^{5}$ the values of $k_1$ and $k_2$ do not vary significantly: we find $0.110< k_1 < 0.124$ and $0.053< k_2 < 0.057$. We also identify a range of values of *s* for which two values of *s* produce the same values of $k_1$ and similarly for $k_2$. Recalling that $k_1$ and $k_2$ are defined such that $\lambda ^{+}(k_1, k_2) = {\bar{\beta }}(v,u)$ and ${\tilde{g}}_2(k_1, k_2) = {\bar{\pi }}_2(v,u,s)$, we find that these regions must be identical, as explained below.

#### Theorem 1

Suppose that *u* and *v* are constant. If $\exists$ $s_1 \ne s_2$ such that $k_1(v, u, s_1) = k_1(v, u, s_2)$, then $k_2(v, u, s_1) = k_2(v, u, s_2)$.

#### Proof

If *u* and *v* are fixed, then since ${\bar{\beta }}$ is independent of *s*, and $\lambda ^+(k_1, k_2) = {\bar{\beta }}(v,u)$, it follows that $\lambda ^+$ is also independent of *s*. Then by (42) and (41),

49 $$\begin{aligned} k_2(v,u,s) = \frac{\lambda ^+ (1 + 2k_1(v,u,s))}{2(k_1(v,u,s)-\lambda ^+)}, \end{aligned}$$

from which we see that

50 $$\begin{aligned} k_1(v, u, s_1) = k_1(v, u, s_2) \implies k_2(v, u, s_1) = k_2(v, u, s_2), \end{aligned}$$

and so the claim holds. $\square$

The reverse of this result for the case where $s_1 \ne s_2$ are such that $k_2(v, u, s_1) = k_2(v, u, s_2)$ can be proved in a similar fashion.

### Full Simulations

Having shown that a single set of ODE parameters can, for particular pairs (*v*, *u*), correspond to two different values of *s*, the initial cell phase ratio, we now turn to look at the dynamics of the PDE model. As an example, we fix $v = 1/16$ and $u = 1/28$, and find two values of *s*, denoted by $s_1 < s_2$, such that $k_1(v,u,s) = 0.11455$ and $k_2(v,u,s) = 0.05541$. We will use these different values of *s* to induce different initial conditions for the ODE model. For each value of *s*, the corresponding initial conditions for the ODE and PDE simulations are

51 $$\begin{aligned} G_1(0) = \frac{1}{1 + s}, \hspace{0.5cm} G_2(0) = \frac{s}{1+s} \end{aligned}$$

and

52 $$\begin{aligned} p(\phi ,0) = {\left\{ \begin{array}{ll} \frac{2}{1 + s}, \hspace{0.5cm} \text { if } \phi \le 0.5 \\ \frac{2s}{1 + s}, \hspace{0.5cm} \text { if } \phi > 0.5, \end{array}\right. } \end{aligned}$$

respectively.

**Fig. 9:**
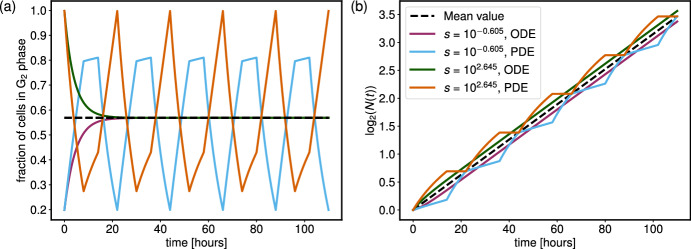
Time-evolution of the proportion of cells in the G$_2$ phase (a), and the total number of cells (b) for two PDE-ODE pairs display different growth dynamics due to different initial phase proportions. Each PDE simulation has $u = 1/28$, $v = 1/16$, while each ODE simulation has $k_1 = 0.11455$ and $k_2 = 0.05541$. The value of *s* determines the initial phase ratio, where we have 1 cell in total initially (so $N(0)=1$). See text for the definition of Mean value.

In Figure 9, we plot the dynamics of the G$_2$ phase proportions and the total cell number density over a period of approximately 120 hours for two pairs of PDE and ODE parameters. In the PDE simulations we take $u = 1/28$, $v = 1/16$, $s_1 = 10^{-0.605}$ and $s_2 = 10^{2.645}$, while in both ODE simulations, we use $k_1 = 0.11455$ and $k_2 = 0.05541$.

In Figure 9(a), we plot the mean G$_2$ proportion for PDE oscillations over a single cell cycle (22 hours), found using (30). In Figure 9(b) we plot the cell density growth curve for a cell population growing exponentially at rate ${\bar{\beta }}(u = 1/28, v = 1/16)$, starting from a single cell. In each panel, these are presented as the “Mean value" curves.

Figure 9(a) shows that the ODE solutions settle to a steady state after one cell cycle. This steady state is identical to the mean of the PDE oscillations, by construction. For the PDE oscillations, both values of *s* lead to oscillations with large peak-to-peak amplitudes, *A* (which we will refer to as just the “amplitude" from now on); when $s = 10^{-0.605}$, $A = 0.85 - 0.2 = 0.65$, and when $s = 10^{2.645}$, $A = 1.00 - 0.25 = 0.75$. We also note that the oscillations appear to be “out of phase" with each other. The maximum amplitude of the oscillations when $s = 10^{2.645}$ occurs at the same time as the minimum amplitude of the oscillations when $s = 10^{-0.605}$. However, the maximum amplitude of the oscillations when $s = 10^{-0.605}$ and the minimum amplitude of the oscillations when $s = 10^{2.645}$ are not aligned. Analytical manipulation of the expressions for $\pi _2(t)$ given by (18)-(24) demonstrates that the extreme values of a given oscillatory solution occur every $\eta _1$ hours, starting from either $t = 0$, 1/2*v* or 1/2*u*. Here, the maximum amplitude of the $s = 10^{2.645}$ case and the minimum amplitude of the $s = 10^{-0.605}$ case occur at integer multiples of $\eta _1$. However, the minimum amplitude of the $s = 10^{2.465}$ case occurs every $\eta _1 = 22$ hours, starting from $t = 1/2v = 8$ hours, whereas the maximum amplitude of the $s = 10^{-0.605}$ case occurs every 22 hours, starting from $t = 1/2u = 14$ hours. This difference will be important when we consider the effect of treatment on these models, as the time in the cell cycle at which treatment is applied may greatly impact subsequent tumour reduction.

This result is lost in the ODE case, where the proportions settle to a steady state, suggesting that the time of treatment application within a single cell cycle would have no effect on treatment efficacy. This also highlights the importance of the initial condition of the PDE model, and the identifiability of the parameter *s*, which we have shown to take two distinct values in some regions of parameter space. If each value of *s* in Figure 9 corresponds to data from a different patient, we see that the optimal time of treatment application within a single cell cycle differs for each patient due to the periodicity that propagates the differing initial conditions. Thus, in situations in which some oscillatory behaviour is seen in cell cycle proportions over time, more insight may be gained regarding optimum treatment plans when the PDE model is implemented.

When looking at the total number of cells in Figure 9(b), we note that the (ODE, PDE) solutions lie either side of the mean PDE curve. The solutions for $s = 10^{-0.605}$ lie below the mean curve, and the solutions for $s = 10^{2.645}$ lie above it. This is because when $s = 10^{-0.605}$ the initial cell density is concentrated in the first phase of the cell cycle, and so it will take longer for the cells to double when compared to the case with $s = 10^{2.645}$, where the initial cell density is concentrated in the second cell cycle phase. Whilst the exponential growth of the ODE solutions prevents the curves from intersecting the mean growth curve, the PDE solutions intersect the mean growth curve every 22 hours (corresponding to the end of each complete cell cycle), demonstrating that the PDE curves generate the same cell numbers as the simple mean growth curve at the end of a complete cell cycle. At intermediate times, regions of slower and faster growth push the PDE solution away from the mean curve.

Without any knowledge of the cell phase proportions as in Figure 9(b), the growth curves of the PDE model provide insight into the underlying cell cycle. The periods of slower growth suggest times at which the proportion of cells in the G$_2$ phase is less than when the population is experiencing a faster growth rate. In this case, where we know the parameter values, this can be explained by the fact that the periods of slower growth correspond to most of the cells being in the G$_1$ phase, and so fewer cells are passing through the end of the G$_2$ phase in these periods. In general, we know from (32) that $N'(t)$ is piecewise constant in time, with exactly two different growth rates occurring within a single cell cycle, namely for $t \in [K\eta _1, (K+1)\eta _1]$. From this, we find that if the initial growth rate is larger than the second growth rate occurring before $t = \eta _1$, then we must have that

53 $$\begin{aligned} \frac{p_0}{L_1} < \frac{sp_0}{L_2}. \end{aligned}$$

Thus, knowing the initial cell distribution via the parameter *s* allows us to deduce an inequality for the relationship between the two cell cycle phase lengths.

In contrast, the ODE population growth curves give little information about the underlying cell cycle parameters. Hence, total population data alone from the PDE model can provide insights into the cell cycle distribution, whereas the ODE model may not.

### Amplitude of Oscillations in Cell Phase Proportions

As the phase proportions in the ODE model settle to a steady state, we choose the peak-to-peak amplitude of the oscillations in the phase proportions as a measure of how well the ODE model fits the PDE model. Thus, for a given PDE parameter set, the corresponding ODE model will be considered a better fit if the PDE displays small oscillations in the phase proportions.

Periodicity in ${\bar{\pi }}_2(t)$ allows us to restrict the amplitude measurements to a single cell cycle. By minimising and maximising these expressions (see equations (18), (20), (22) and (24)) with respect to time for $t \in [0, \eta _1]$, we can calculate the amplitude of the oscillations analytically (results not shown for brevity).

**Fig. 10:**
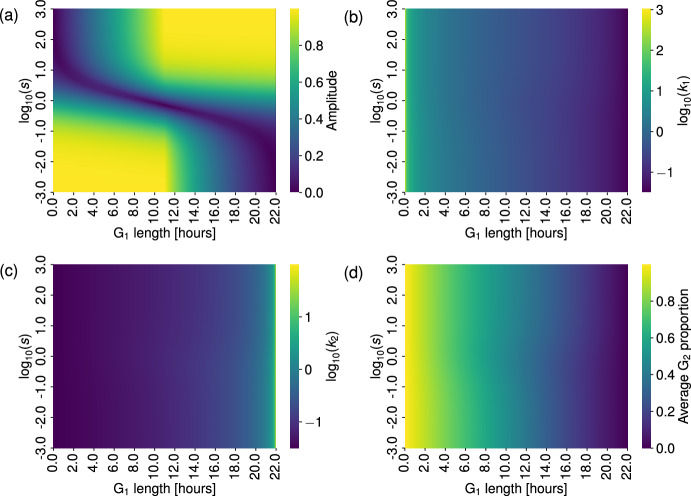
The relative length of each cell cycle phase largely controls both the PDE and ODE dynamics. Plots of (a) the amplitude of the G$_2$ oscillations in the PDE model, (b) the value of ODE parameter $k_1$, (c) the value of ODE parameter $k_2$ and (d) the average proportion of cells in the G$_2$ phase from the PDE model. In each case, we fix $\eta _1 = 22$ hours, and vary the length of the G$_1$ phase (1/2*v*) and the initial cell distribution factor *s*

As before, we fix the cell cycle length so that $\eta _1 = 22$ hours, which restricts *v* and *u* so that $1/v + 1/u = 44$. Figure 10 displays four heatmaps for the peak-to-peak amplitude as we vary the initial proportion constant *s*, and the length of the G$_1$ phase, thus fixing the length of the G$_2$ phase. As discussed in Section 4.3, the cell cycle length and phase lengths are known to vary between different cell lines. Therefore, Figure 10 is intended to represent results from the PDE model and ODE model under different cell lines, each with the same total cell cycle length of 22 hours but different phase lengths within that. Figure 10(a) shows the amplitude of the G$_2$ proportion oscillations, and we see that we have two distinct regions of parameter space where the amplitude is high ($> 0.7$), namely when G$_1$ is less than 11 hours and most of the initial cells are concentrated in G$_1$, and when the length of G$_1$ is greater than 11 hours and most of the initial cells are concentrated in G$_2$. In the first case, we find that having most of the cells initially concentrated in G$_1$ means that the G$_2$ proportion is initially low, whilst the short G$_1$ phase length allows this majority to quickly pass to G$_2$, causing the cells to transiently congregate in the G$_2$ phase, leading to a large G$_2$ proportion. Thus, it is to be expected that this case leads to a large G$_2$ oscillation amplitude. A similar argument explains the second case. In contrast, we find that as we increase the length of the G$_1$ phase, the initial cell distribution required to keep the oscillatory amplitude small ($<0.2$) leans more towards an initial G$_1$ majority. This is to be expected, as *s* is defined to be the ratio of the number of cells initially in the G$_2$ phase to the number of cells initially in the G$_1$ phase. For a large value of *s*, the cells are initially largely concentrated in G$_2$, and so the G$_2$ proportion is initially high. With a small G$_1$ length (and correspondingly, a large G$_2$ length) we expect the proportion of cells in G$_2$ to remain high throughout, as the small proportion of cells that pass into G$_1$ at any given time will quickly progress back to G$_2$, thus leading to a small amplitude. As the length of the G$_1$ phase increases, we would expect that we would need the balance of the initial cell distribution to shift to being initialised in the G$_1$ phase in order to reverse this result.

Figure 10(b) and 10(c) display how the ODE parameters $k_1$ and $k_2$ change over the same parameter space. As to be expected from the sensitivity analysis in Figure 6, we see that any variations are largely independent of the value of *s*. Finally, in panel (d), we present the average proportion of cells in the G$_2$ phase, ${\bar{\pi }}_{2}$, over this parameter space. Again, this value is largely independent of *s*, and decreases with an increasingly long G$_1$ phase.

Thus, we see that for a fixed cell cycle length and a given value of *v*, the initial cell distribution factor, *s*, in the PDE model has little effect on the corresponding ODE parameters and approximately fixes the average of the G$_2$ proportion oscillations (and the steady-state of the corresponding ODE model). However, the parameter *s* leads to large variations in the oscillation amplitude of the PDE model, and therefore how well the ODE model captures its dynamics. For example, for a small value of *v* (a long G$_1$ phase), the long-time behaviour of the ODE model will predict that the G$_2$ proportion settles to a small value, but the PDE oscillations about this value will have a large amplitude if most cells are initially in the G$_2$ phase, and a small amplitude if the initial cells are instead focused in the G$_1$ phase. In this case, the ODE model approximates the PDE dynamics well when *s* is small, and the approximation is poor when *s* is large.

Such considerations of the oscillatory amplitude are also important when investigating how treatment may affect the cell population. When using the ODE model to represent cell populations, the rapid approach to steady-state cell cycle phase proportions suggests that after an initial transient, the effect of a cell-cycle phase-dependent treatment will be independent of the time at which it is applied. In contrast, the periodicity of the PDE cell cycle phase oscillations means that treatment efficacy could depend significantly on the time at which it is applied.

For example, radiosensitivity is highest for cells in the $G_2/M$ phases of the cell cycle (Pawlik and Keyomarsi 2004), and so if the PDE model is used to represent the cell cycle, the timing of the treatment application should be optimised to ensure that the proportion of cells within these phases is at its highest at the start of treatment. Time-course data of cell cycle phase proportions of in vivo tumours are difficult to obtain, and so any experimental data are largely from in vitro models. Even in this simple PDE model, we see that all three parameters *v*, *u* and *s* affect the oscillatory amplitude and period, and as such, predicting this amplitude for experimental results without detailed time-course data is likely to prove difficult.

Further, one effect of radiotherapy is to slow cell cycle progression and cause cell accumulation in the G$_2$ phase post-treatment, which is dose-dependent and induces oscillatory dynamics in the cell cycle phase proportions (Pawlik and Keyomarsi 2004; Geldof et al 2003). As such, the PDE model presented here may be a useful and simple tool for modelling this accumulation, as well as for obtaining the optimal height of this G$_2$ accumulation, at which point further radiotherapy can be applied, as considered experimentally by Geldof et al (2003).

**Fig. 11:**
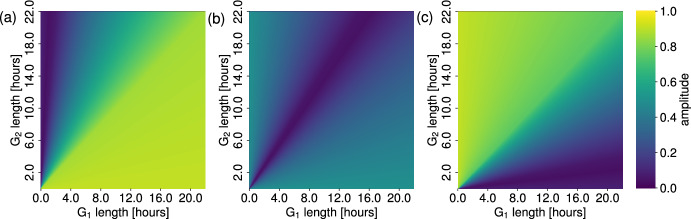
The relative cell cycle phase lengths, and total cell cycle length, modulate the amplitude of the PDE oscillations in an initial phase distribution-dependent manner. Plots of the oscillatory amplitude in the G$_2$ phase proportion as the length of the G$_1$ and G$_2$ phases vary, for three different initial distribution factors, where (a) $s = 10$, (b) $s = 1$ and (c) $s = 0.1$.

Figure 11 shows how the amplitude of the G$_2$ phase proportion varies as the lengths of the G$_1$ and G$_2$ phases vary, for a decreasing initial proportion of G$_2$ cells from left to right. For example, fixing the length of the G$_1$ phase and increasing the length of the G$_2$ phase has an initial-condition dependent effect on the resulting amplitude of the oscillations. Once again, the initial conditions used in our simple PDE model are likely too simplistic to represent a feasible cell cycle distribution, and the effects of more complex initial distributions are not investigated further here. However, we see that this simple model presents possibilities for future investigations using more complex functional forms and explicit applications of treatment.

This gives some indication as to how the oscillatory amplitudes change if one of the phases is shortened or elongated by anti-cancer treatment, such as the cell cycle arrest and reduced proliferation rate induced by radiotherapy (Carlos-Reyes et al 2021). Recent work has also shown clinical success with the use of cyclin-dependent kinase inhibitors (CDKi) to target tumour proliferation (Suski et al 2021; Álvarez Fernández and Malumbres 2020). CDKi have been shown to cause cell cycle arrest (the arrest phase is dependent on the specific CDKi used), thus elongating the length of cell cycle phases, and provides a promising avenue for future experimental and clinical investigation.

### Fitting the ODE Model to PDE Data

Throughout this work, we have constructed our corresponding ODE model for each PDE model under the assumption that we know each of the three PDE parameters, (*v*, *u*, *s*), in order to match the long-term ODE behaviour with the average PDE behaviour. In reality, experimental data can only provide us with snapshots of the behaviour. We now briefly turn our attention to fitting the ODE model to the PDE model without this complete parameter knowledge.

We use our analytical expressions for *N*(*t*) and $\pi _{G_2}(t)$ (equations (17) - (24)) to simulate the PDE model under a given parameter set (*v*, *u*, *s*) at a set of discrete times $(t_1, t_2, ..., t_n)$. This allows us to generate data points from the PDE model that we use as synthetic data for ODE fitting purposes. We then look at how the fitted ODE parameters compare to the “analytically matched" ODE parameters considered until now.

Denoting the two sets of data by $\vec {\pi }_{2} = (y_1, y_2, y_3, ..., y_n)$ for the proportion of cells in the G$_2$ phase at *n* increasing values of *t*, and $\vec {N} = (z_1, z_2, z_3, ..., z_n)$ for the total number of cells at the same values of *t*, we use non-linear least squares to fit these data to the ODE model. We assume that the first data point is taken at $t = 0$, and so we know the value of *s* in the initial conditions for the ODE model. Therefore, we seek to find the set of parameters $\vec {K} = (k_1, k_2)$ for the linear ODE system (35) (subject to initial conditions $G_1(0) = (1-y_1)z_1$, $G_2(0) = y_1z_1$) that best fit the PDE data. We use a non-linear least squares fitting algorithm to fit the solutions of the ODE model for the total cell number and G$_2$ phase proportion to the discrete PDE data points. As we are fitting the ODE model to two different quantities that take values on different scales (the total cell number can vary between 1 and $10^3$ for the chosen parameters, whilst the G$_2$ phase proportion is fixed to lie between 0 and 1), we take a log-transform of the least-squares fitting function. Thus, the best-fit set of ODE parameters $\vec {K}$ is the vector that minimises the loss-function

54 $$\begin{aligned} S_n^{L} = \sum _{k = 1}^{n}(\ln (z_k) - \ln (N(t_k, \vec {K})))^2 + \sum _{k = 1}^n (\ln (y_k) - \ln (\pi _{G_2}(t_k, \vec {K})))^2, \end{aligned}$$

and we use the Python package $\texttt {Pints}$ (Clerx et al 2019) to perform the fitting.

**Fig. 12:**
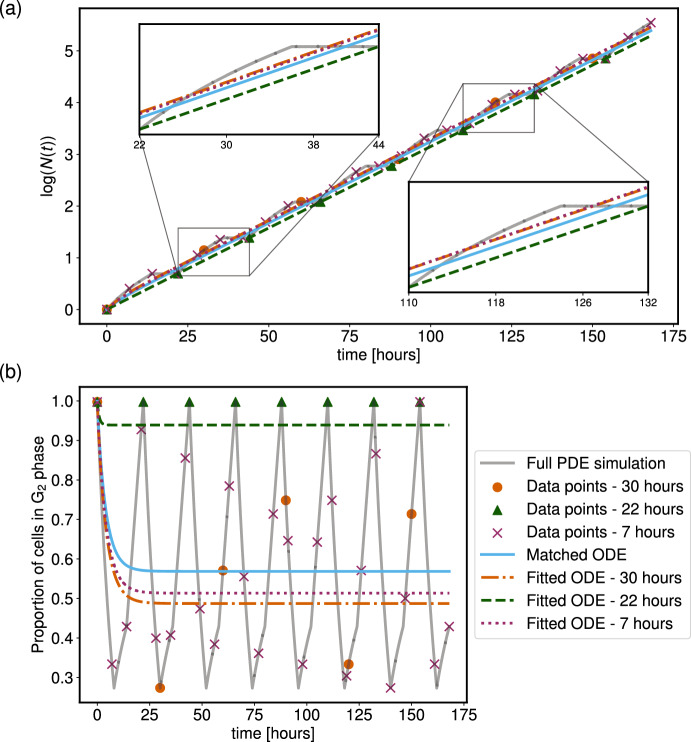
The frequency of data sampling from the PDE impacts the goodness of fit between the fitted ODE and the matched ODE. In each plot, the scattered points represent data from the PDE model (shown in grey), which are used in the least-squares method to optimise the ODE model fit. The data points correspond to the PDE system every 7, 22 and 30 hours for the purple, green and pink points, respectively, with $u = 1/28$, $v = 1/16$ and $s = 10^3$. The dashed lines display the optimal fit to the ODE model using least-squares with the log form for the residuals (see text for details). The blue curve is the solution to the ODE model using the analytical matching process discussed previously. Panel (a) shows the log-transformed population count of the underlying PDE model, and the various fitted and matched ODE systems, and (b) displays the corresponding proportion of cells in the G$_2$ phase.

Figure 12 shows the results of this fitting when the simulated PDE uses parameter values $v = 1/16$, $u = 1/28$ and $s = 10^3$, to mimic a realistic cell cycle duration as in Section 4.3. This large value of *s* is chosen to obtain large oscillations in the G$_2$ proportions, as discussed above, but is otherwise an arbitrary choice. We choose to sample the PDE curves at three different time intervals, namely every 7, 22 and 30 hours. Out of the three sampling frequencies, we find that the 7 hour intervals create the closest ODE fit to the dynamics of the “analytically matched" ODE model, whilst the 22 hour intervals create the worst fit. These three examples demonstrate the importance of the sampling frequency if we assume that the PDE model is the “ground truth".

Whilst the 7 hour sampling produces the closest fit to the “analytically matched" ODE, we find that the 30 hour sampling produces only a slightly worse match. In contrast, the 22 hour sampling produces a fitted ODE model that matches the “analytically matched" ODE very poorly. This can be explained by looking at the location of the data points in Figure 12(b). The length of the cell cycle is 22 hours in this case, and so the periodicity of $\pi _{G_2}(t)$ means that sampling the G$_2$ proportion every 22 hours only records data when the proportion is at its maximum value, and so does not capture the large oscillation in $\pi _{G_2}(t)$. On the other hand, the data points taken every 7 and 30 hours do a much better job of capturing the range of dynamics in the oscillations of $\pi _{G_2}(t)$, and thus create a better representation of the overall dynamics of the underlying PDE.

We repeated this fitting procedure for samples taken every 1 hour, and every 2 hours, and found little difference between the resulting ODE models, and the ODE model fitted to the data sampled every 7 hours (results not shown).

Therefore, we find that if our underlying data have oscillatory behaviour in the cell cycle phase proportions, then the frequency at which we collect the data can impact the range of this oscillatory behaviour that is seen. Thus, when fitting the ODE model to the data, we must be aware that the ODE model might not accurately capture the average behaviour of the underlying oscillations. One possible solution to this may be sampling at irregular intervals in an attempt to capture as much of the underlying oscillatory behaviour as possible, although we refrain from considering this approach here for brevity.

In this section, we have defined a method of “matching" the ODE model to the PDE model via expressions for the key quantities of growth rate and cell cycle proportions. This allows us to obtain an ODE model with a long-term growth rate and cell cycle phase proportions that matches the corresponding average behaviour of a given PDE. Given the analytical expressions for each of these quantities in each type of model, we found that searching for a given set of ODE parameters $(k_1, k_2)$ determined a simple relationship for the PDE parameters (*v*, *u*) by matching the growth rates of the two models. We also saw that the problem is under-specified due to the inclusion of the initial cell cycle phase distribution parameter *s* in the PDE model quantities. We found that this leads to regions of (*v*, *u*) space where it is possible to obtain two different initial distribution factors, *s*, that generate identical ODE parameter sets.

Furthermore, we restricted our PDE parameters (*v*, *u*) to biologically realistic values, and explored the consequences on the derived values of the ODE parameters $(k_1, k_2)$, and how altering the value of *s* can maintain the mean (long-term) growth rate and cell cycle phase proportion for the ODE (PDE) models, yet lead to dramatically different behaviour in the full underlying PDE dynamics.

We also introduced the amplitude of the PDE phase proportion oscillations to represent a measure of agreement between the ODE and PDE models. We found that this amplitude is dependent on each of the parameters (*v*, *u*, *s*) and can be used to consider the impact of treatment such as radiotherapy on a population of cells.

Finally, we considered how, in the absence of exact parameter values to complete the analytical “matching" process between the two models, we can fit the ODE model to a discrete set of synthetic PDE data. This illustrated how the oscillatory behaviour of the PDE model requires careful consideration of the frequency of data collection in order to produce a good fit to the average underlying behaviour by the ODE model.

## Discussion

When modelling the cell cycle mathematically, there is a choice to make regarding whether to place cell populations into well-mixed compartments, each representing a different cell cycle phase, or to consider the cell cycle as a continuum through which a cell progresses at a given velocity. These two views lead to different modelling structures. Here, we analysed the simplest form of the continuum model as a structured PDE, and then compared the insights gained to those from a well-mixed compartment-based ODE system.

In Section 2 we introduced the PDE model, taken from work by Rubinow (1968) and specified simple functional forms for the cell cycle progression velocities and initial conditions. Integration of the solution produced expressions for the proportion of cells in each of the two considered phases and the population growth rate as functions of time. We found that these quantities are periodic with period equal to a single cell cycle. Further integration to average these results over the period generated expressions for the average growth rate and proportion of cells in the G$_2$ phase as functions of the progression velocities (*v*, *u*), and the relative number of cells initially in each compartment, *s*.

Linear compartment-based ODE systems that represent progression through the cell cycle often have cell phase proportions that settle to a stable steady-state, and population growth rates that are dominated by the largest eigenvalue of the linear system. This provides an avenue for matching the averaged results of the PDE system to the long-time behaviour of a corresponding linear ODE system. In Section 3, we defined this linear ODE system, and solved for the analytical expressions of this steady-state and long-term growth rate in terms of the model parameters.

In order to investigate cases in which the PDE model produced additional insight into the cell cycle that could not be captured by the ODE, Section 4 focused on relating and comparing the two model systems. We started by linking the model parameters together via the growth rates and G$_2$ cell proportions so that the long-time ODE dynamics represent the average of the oscillatory PDE dynamics. Further consideration of these relationships included a sensitivity analysis, from which we found that the parameters controlling G$_1$ progression in each model were closely linked, with the same result for the G$_2$ parameters.

The inclusion of a simple uniform-density initial condition in the PDE model caused the parameter matching to be under-specified, from which we found that two different initial configurations of cell-cycle distribution in the PDE model could generate a single set of “averaged" ODE system parameters. As the long-time ODE behaviour considered here is independent of the initial conditions, perfectly matching the mean of the PDE results to the long-time ODE results still leads to identifiability issues with regards to the initial conditions of both the PDE (we may have two possible values of *s*) and the ODE (we gain no information about the initial cell distribution). These two PDE parametrisations have distinct growth dynamics, both in the amplitude of cell cycle proportion oscillations, and the overall population growth rate at a given time. When simulating both the PDE and ODE models for a given matched parameter set, we find that the PDE can produce more complex dynamics for the cell cycle distribution and growth rates at a given time via the oscillatory behaviour inherent in the model.

As discussed throughout, many cancer treatments have cell-cycle specific effects on cells. We have seen here that in a cell population experiencing non-monotonic fluctuations in the proportion of cells in a given cell cycle phase, the simple two-compartment ODE model is incapable of capturing these fluctuations. On the other hand, the structured PDE model captures these oscillations fully, even using the simplest piecewise-continuous functional forms for the initial conditions and cell cycle progression velocities. Therefore, the PDE model is better equipped for considering the effects of these cell-cycle specific treatments in these fluctuating cases. This demonstrates that for parameter sets leading to large PDE oscillatory amplitudes, the mean is not a good measure of the system behaviour, and as such the ODE model provides a poor approximation in the context of treatment application.

Further work may consider the effects of imposing explicit treatment on the PDE model in a cell-cycle specific manner. We have found that the time at which treatment is applied in the PDE model has the ability to have significant impact on treatment efficacy due to the range of cell phase proportion amplitudes possible in the parameter space considered here. Hence, it may be interesting to find analytical results for cell population reduction under treatment application at different times along a single period of phase proportion oscillation, and investigate the range of different outcomes predicted by this model. This could be accomplished by the addition of a death term of the form $-\lambda (\phi ,t)p(\phi ,t)$ to the right-hand side of equation (1), as an extension to the time-independent death rate $-\lambda (\phi )$ in  Rubinow (1968). Whilst we have seen here that the cell-cycle fluctuations present in the PDE model cannot be captured by the ODE model, it would be interesting to investigate how the averaged effects of treatment on the PDE model compare to the results of treatment in the ODE model. For example, should we still expect the long-term ODE model behaviour to represent an average of the PDE model? This would be made more complex if treatment is assumed to be effective over a window of time. For example, if the treatment is effective for a sufficient amount of time after initial application, could it be possible that results predicted by the PDE model have closer agreement with those from the ODE model due to the treatment being effective for a larger portion of a single PDE oscillation?

Another extension to this comparison would be to consider a system of non-linear ODEs, rather than a linear model. By assuming, for example, that the progression rates between phases depend on population density and/or intracellular protein levels (Falcó et al 2025; Adam 1980; Tyson and Novák 2001, 2022), we could provide a more realistic description of the biology within an ODE framework. In addition, we could derive a non-linear ODE system from the PDE system presented here. Both approaches are likely to be more faithful to the underlying biology, but it would be more challenging to compare them to PDE models using analytical methods.

Other future work could focus on the identifiability of the PDE model (Wieland et al 2021; Renardy et al 2022). Given the analytical tractability of the simple PDE model presented here, a natural next step would be to consider the structural identifiability of the parameters (*v*, *u*, *s*) using the measurable outputs of cell phase proportions and total cell density. Furthermore, given the oscillatory behaviour of the cell-cycle proportions resulting from this PDE model, we could also assess the practical identifiability of the parameters by trying to ascertain the parameter values from noisy data.

Following from this linking of the two model types, further work could also focus on fitting the PDE model to experimental data. As mentioned previously, this has been considered for a variety of cell cycle models, including Tyson et al (2012); Gabriel et al (2012); Ubezio (2024); Celora et al (2022). More specifically, work by Tyson et al (2012) and Gabriel et al (2012) consider data from single cells which quantify their inter-mitotic time, and as such, their methods rely on having the detailed data on the scale of individual cells. However, such work would provide an excellent framework for studying the impact of stochasticity of inter-mitotic times in individual cells. When considering cell cycle progression for a population of cells, studies find that cells desynchronise and populations eventually settle to have steady proportions within each phase (Nowak et al 2023). Work by Vittadello et al (2019) also shows oscillatory behaviour in the ratio of the number of cells in one cell cycle phase compared to the other phases during the first few cell cycle lengths. An ODE model consisting of many compartments is proposed and fitted to these data with success. Mathematical models of this initial synchronised oscillatory behaviour followed by a formation of steady cell proportions have been proposed, including both stochastic models (Olofsson and McDonald 2010) and deterministic structured-PDE models (Chiorino et al 2001). This deterministic structured-PDE model assumes that a cell of given age $a>0$ divides at a rate determined by a probability density function for the cell cycle length. Whilst capturing both the initial oscillatory behaviour and later steady behaviour, the analysis of the PDE model is not analytically tractable.

Therefore, whilst the ODE model considered here might be better at capturing the long-time behaviour of a cell population, the fit of the structured PDE model to the oscillatory data collected by Vittadello et al (2019) could be considered. Capturing a good fit using this simple PDE model would allow for analytical tractability when modelling these early oscillatory stages of population growth. Obtaining a good fit to these data may require a more complex form of the initial distribution (though perhaps one that still produces analytically tractable results) than the piecewise-constant form used here, and so we leave this for future work.

## Data Availability

Not applicable

## Declarations

### Code availability

The code used to generate the figures in this publication can be accessed by contacting ruby.nixson@maths.ox.ac.uk.
